# Surgical Approaches to Retinal Gene Therapy: 2025 Update

**DOI:** 10.3390/bioengineering12101122

**Published:** 2025-10-20

**Authors:** Milin J. Patel, Sohum Sheth, Jessica Mar, Ninel Z. Gregori, Jesse D. Sengillo

**Affiliations:** 1 Department of Ophthalmology, Bascom Palmer Eye Institute, University of Miami Miller School of Medicine, Miami, FL 33136, USA; 2 Philadelphia College of Osteopathic Medicine, Suwanee, GA 30024, USA; 3 University of Florida College of Medicine, Gainesville, FL 32610, USA

**Keywords:** retina, gene therapy, subretinal, suprachoroidal, intravitreal, inherited retinal disease, age-related macular degeneration

## Abstract

Gene therapy offers a promising new frontier in the treatment of inherited and acquired retinal disease. This review describes the current surgical delivery approaches for gene therapy to the retina—subretinal, suprachoroidal, and intravitreal—and provides an update on the state of the art for each method in 2025.

## 1. Introduction

**Figure 1:**
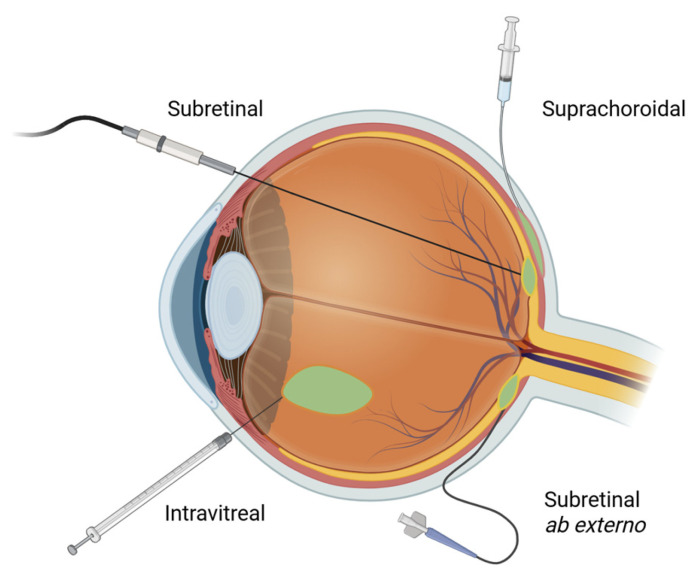
Surgical approaches to retinal gene therapy.

Gene therapy is a rapidly evolving field that aims to treat or prevent disease by introducing, modifying, or silencing genetic material within a patient’s cells. The human eye is an ideal target for gene therapy due to its unique anatomical, physiological, and immunological characteristics. Its small, compartmentalized structure allows for precise delivery of therapeutic vectors in low volumes, minimizing systemic exposure and reducing the risk of adverse effects [1]. The eye is also a relatively immune-privileged site, meaning it has a reduced immune response to foreign antigens, which lowers the risk of inflammation to gene therapy vectors [2]. The retina, in particular, is accessible through a variety of minimally invasive surgical approaches [Figure 1]. The ability to noninvasively monitor retinal structure and function using such imaging modalities as optical coherence tomography (OCT), widefield imaging, fluorescein angiography (FA), and fundus autofluorescence (FAF) further facilitates assessment of treatment efficacy and safety in clinical trials and routine practice.

Traditional gene therapy delivers functional copies of defective genes directly to retinal target cells, typically using adeno-associated viral (AAV) vectors. Adeno-associated viral vectors are the most commonly utilized viral vectors due to their high stability and long-lasting expression—existing as episomal DNA in non-dividing cells, such as photoreceptors (PRs) and retinal pigment epithelium (RPE) [3]. Other vectors include adenoviral, lentiviral, and retroviral vectors [Table 1]. Adenoviral vectors were the first to be evaluated for retinal gene transfer [4]. These non-integrating vectors can transduce a broad range of dividing and non-dividing targets and can carry large genetic payloads up to 30 kilobases (kb) [5]. They exhibit quick onset but rapid clearance due to strong innate and adaptive immune responses [5]. Adeno-associated virus (AAV) vectors are non-integrating and non-replicating in nature and exhibit weak immunogenicity; however, they have a lower packaging capacity around 4.7 to 5.0 kb [5]. Lentiviral and retroviral vectors offer similar advantages to AAVs but larger delivery capacities than AAVs; however, their integration into the host genome raises concerns for off-target insertion and mutagenesis [5]. Nonviral vectors including direct injection of naked DNA and RNA and nanoparticles have also been studied; however, implementation into clinical trials has been difficult due to challenges with stability and degradation [3].

**Figure 2:**
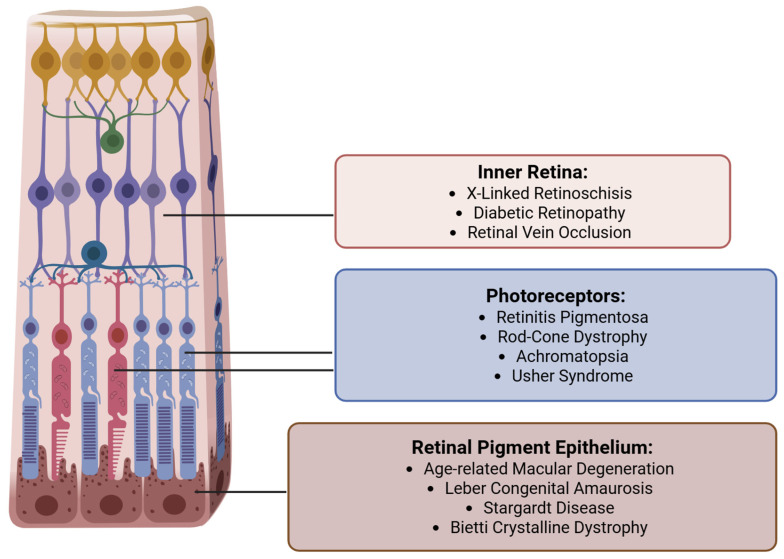
Select retinal diseases and accompanying gene therapy targets.

**Figure 3:**
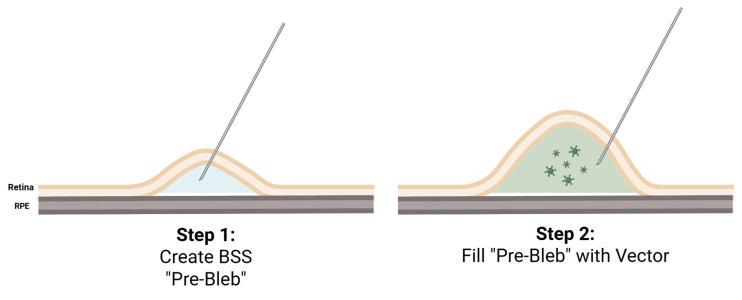
“Two-Step” Subretinal Injection Method.

Targeted retinal gene therapy has shown promise in halting or even partially reversing vision loss in certain acquired and inherited retinal diseases (IRDs) [9] [Figure 2]. A landmark product is voretigene neparvovec-rzyl (Luxturna, Spark Therapeutics, Inc.), the first FDA-approved gene therapy for a retinal condition, which treats patients with biallelic RPE65 mutations causing Leber congenital amaurosis (LCA) or early-onset retinitis pigmentosa (RP) [8]. Other inherited retinal diseases currently targeted for retinal gene therapy include various forms of retinitis pigmentosa and LCA, achromatopsia, choroideremia, Usher Syndrome, Bietti Crystalline Dystrophy, Stargardt Disease, and X-linked retinoschisis [Figure 3]. Beyond monogenic disorders, gene therapy is also being applied towards acquired retinal diseases such as age-related macular degeneration and diabetic retinopathy, where sustained intraocular production of anti-VEGF proteins through gene delivery could reduce treatment burden [6].

Retinal gene therapy is delivered through three primary routes—subretinal (~57%), intravitreal (~37%), and suprachoroidal (~3%) [Figure 1]—each with distinct advantages and disadvantages [7]. The choice of delivery route largely depends on disease indication and the neutralizing effect of ocular barriers. There are several physiological barriers that influence the efficiency and transduction potential of retinal gene therapy delivery. The internal limiting membrane (ILM) is a basement membrane on the innermost side of the retina that acts as a physical and biochemical barrier that impedes vector diffusion [10]. The vitreous itself may also act as a diffusion barrier for injected vectors. This is a distinct disadvantage of intravitreally delivered vectors, which are bypassed by subretinal and suprachoroidal approaches. The outer blood–retinal barrier (BRB) is another obstacle formed by the tight junctions between RPE cells that limit the diffusion of vectors from the choroid into the retina. Intravitreal and subretinal injections bypass the BRB, while suprachoroidal approaches must overcome this hurdle to reach the PRs effectively. These shortcomings must be balanced with the safety and invasiveness of each delivery method. For example, while subretinally delivered gene therapy offers localized and precise delivery which bypasses most anatomical barriers, it is the most invasive and surgically demanding approach. Conversely, while intravitreal injections are minimally invasive and can be performed in clinic, they encounter the most ocular resistance in reaching the outer retina in the form of vitreous dilution, neutralizing antibodies, and the ILM. Newer viral vectors are being designed to overcome these challenges.

The field of retinal gene therapy is advancing at a remarkable pace, driven by breakthroughs in vector design, improvements in existing delivery methods, utilization of ancillary technology (i.e., intraoperative OCT) and development of novel surgical approaches (robotics, microinjectors). This review aims to provide an updated review of the three major delivery approaches to retinal gene therapy: subretinal, suprachoroidal, and intravitreal. Unlike earlier reviews, this article integrates the most recent preclinical and clinical studies over the past 3 years into 2025. This review integrates practical surgical insight with emerging evidence from these studies: highlighting novel advancements, practical applications, and limitations of each surgical approach. A thorough grasp of current developments and techniques in retinal gene therapy informs future research directions and leads to a more contextual understanding of this evolving field for clinicians and academics alike.

## 2. Methods

Select publications were filtered using Google Scholar and the PubMed index. The studies included in our review of recent developments were filtered from January 2022 to May 2025. Articles published prior to 2022 were included primarily for contextual discussion. Current clinical trial data was acquired from ClinicalTrial.gov. We also included relevant presented abstracts related to ongoing clinical trial results. Illustrations were created with BioRender.

## 3. Subretinal Delivery

### 3.1. Background

Direct subretinal delivery has become the preferred approach for the majority of gene therapies targeting the outer retina and RPE. It is heavily utilized in ongoing retinal gene therapy clinical trials. The subretinal space (SRS) is the area between the neurosensory retina—specifically, the photoreceptor outer segments—and the RPE. The SRS plays a critical role in phototransduction, retinal adhesion, and nutrient exchange [11] and is a key site of degeneration in many IRDs and in age-related macular degeneration (ARMD). Delivery of therapeutics to this space allows direct treatment of cells affected in photoreceptor and RPE diseases, bypassing the ILM and the blood–retinal barrier. This is particularly important in gene therapy, as many vectors require direct exposure to cellular targets for efficient transduction [10]. Injection of agents in the SRS creates an enclosed pocket or “bleb”, which limits distribution of the therapeutic agent. This confinement, while limiting the treated area, minimizes the risk of off-target effects and systemic exposure and leads to increased concentration of the vector near the cells of interest [12]. The immunogenicity of vectors delivered through a subretinal approach is also reduced by the relative immune-privileged nature of the SRS. A combination of protection from the BRB [13], immunosuppressive cytokines from resident microglia [14], immunomodulatory properties of RPE [15], and apoptotic defense mechanisms maintain this immune tolerance. Lastly, widespread familiarity with standard three-port pars plana vitrectomy (PPV) has facilitated adaptation of existing surgical technique to subretinal vector delivery protocols, with transvitreal subretinal delivery being the most common approach utilized in most current gene therapy trials. In this section, we describe the surgical approach of subretinal gene therapy delivery. We then discuss recent developments in the subretinal approach, including optimization of the existing transvitreal technique, robot-assisted subretinal delivery, and novel non-vitrectomized subretinal approaches.

### 3.2. Current Subretinal Gene Therapy Delivery Technique

Currently, therapeutic delivery to the subretinal space has primarily been performed via a standard 23- or 25-gauge three-port PPV and creation of a subretinal pocket or a “bleb” between the neurosensory retina and the RPE where the viral vector is injected [Figure 3]. Perioperative treatment with systemic corticosteroids is typically used to modulate immunologic responses that may be triggered by viral vectors, and some trials also employ periocular steroids. Routine topical steroids with adjustment based on the intraocular inflammation present are also typical, along with routine postoperative topical antibiotics [14].

Triamcinolone acetonide is typically used to enhance visualization of the vitreous [16]. A posterior vitreous detachment (PVD) is achieved across the posterior pole to allow for retinal penetration with a 38- or 41-gauge subretinal cannula, which is most commonly performed near the major vascular arcades [17]. Lifting the hyaloid from peripheral retina is not critical, and excessive traction must be avoided to minimize the chance of a retinal break within thin and degenerated retina. Frequently, the peripheral vitreous is very adherent and trimming it without lifting it off the retina is sufficient in most cases.

To elevate the neurosensory retina from the RPE, some trials utilize an initial injection of balanced saline solution (BSS) to create a so-called pre-bleb, particularly if the retina is tightly adherent as may be seen with many IRDs [17]. This helps ease the injection of the viral vector and avoid running out of the gene product, especially if the amount of the provided therapeutic agent is small. The amount of BSS injected is typically small, approximately 30 microliters [17]. The retinotomy is typically performed along the major retinal arcades near an easily recognizable vascular landmark to allow cannula re-entry [6,18]. The therapeutic vector is then slowly injected through the same retinotomy into the BSS pre-bleb under foot-pedal control in what is known as the “two-step” approach [Figure 3]. A “one-step” approach, without the BSS pre-bleb, is also utilized if the amount of viral vector provided is large and the retina is not particularly adherent [19]. The typical amount of viral vector injected into a bleb is between 50 and 300 microliters. The infusion cannula pressure is typically reduced to 10 or 20 mmHg during the injection to ease the delivery of the viral product. The proposed benefits of the one-step approach include a decreased risk of accidental secondary retinotomy and a decreased risk of vector reflux due to the lack of a second re-entry into subretinal space [19].

**Figure 4:**
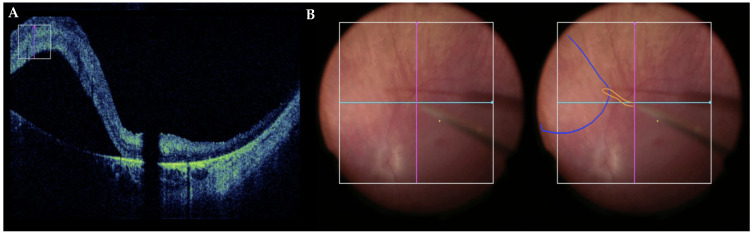
(**A**) Visualization of the subretinal bleb with intraoperative OCT. A shadow created by the subretinal cannula is seen. (**B**) En face view of subretinal bleb (blue overlay) and extendable small gauge subretinal cannula (orange overlay) through microscope-integrated heads-up display. The pink and sky-blue overlays represent the vertical and horizontal axis, respectively.

Subretinal delivery of vector and bleb formation can be monitored and confirmed with microscope-integrated intraoperative optical coherence tomography (OCT) and has been shown to improve safety and precision of subretinal delivery [12,20] [Figure 4]. The OCT allows for real-time monitoring of fluid entering subretinal space—thus avoiding suprachoroidal injection—and careful observation of the fovea. After the completion of subretinal injection, irrigation of the vitreous cavity with BSS or air–fluid exchange is performed to remove any refluxed virus and minimize the risk of postoperative inflammation [12,17].

Disadvantages of the transvitreal subretinal approach largely include potential for iatrogenic retinal break, macular hole formation, endophthalmitis, increased intraocular pressure, nuclear cataract formation [21], reflux of viral vector [22], chorioretinal and RPE atrophy [23,24], subretinal deposits [25], and iatrogenic choroidal neovascularization [26].

### 3.3. Optimizing the Pars Plana Vitrectomy Approach

As subretinal gene therapy advances in complexity and scope, refining the existing PPV techniques remains an important step in ensuring consistent, reproducible, and safe surgical outcomes. Proper creation of the subretinal bleb is a critical determinant of success in subretinal gene therapy. Pedal control—as opposed to manual injection by an assistant surgeon—provides far superior control of the injection pressures and speed [16,27]. Based on our extensive experience with various IRDs and ages of patients, the ease with which the bleb is raised and the maximum injection pressure required to enter subretinal space vary greatly among patients. A number of recent reports have focused on this crucial step of subretinal vector delivery, and there is growing evidence that variations in surgical technique—including the choice between manual injection versus microinjector-assisted delivery [27], the speed and pressure of injection [27], and prior ILM peeling [28]—can affect efficacy and/or the degree of iatrogenic damage to the retina and RPE during subretinal delivery. The MedOne microinjector used currently contains an adaptor that allows its tubing to connect to the viscous fluid control (VFC) function on the vitrectomy machine (the function commonly used for silicone oil injection), and the VFC injection pressure is typically set between 10 and 18 psi. The injection pressure is a critical factor that surgeons can control in order to make the subretinal delivery safer. In monkey eyes, subretinal injection of BSS at 20-psi was shown to lead to IS/OS defects, such as thinning of the outer segments and thickening/multilayering of RPE at 1 week after the procedure. Although these changes completely resolved 5–6 weeks later, avoiding similar effects in humans is desirable [29]. A study by Olufsen et al. demonstrated significantly higher outer retinal and RPE damage scores at 32 psi compared to 14 psi injection rates using a controlled subretinal injection system in live porcine retinas [27]. Setting the VFC injection pressure at the lowest setting necessary to achieve a steady drip of fluid and only raising it if truly unable to raise a bleb will help minimize the injection pressure used. Beveling the subretinal cannula at 45 degrees may also be utilized to ease retinal penetration without going to excessively high injection pressures [16]. Applying only light pressure against the inner retina while attempting to raise the bleb will help avoid occlusion of the fine subretinal cannula and allow the fluid to penetrate the retina.

A recent study by Scruggs et al. reported that pre-blebs generally required greater injection pressures compared to the propagation of the retinal bleb. Thus, reducing the maximum VFC injection setting or reducing the pressure on the pedal during viral vector injection in the two-step approach would also be prudent. In that study, younger patients generally required higher injection pressures during pre-bleb creation [30]; thus, the surgeon must be cognizant of the interplay between the age of the patient and potential increased risk of RPE damage [27]. Internal limiting membrane peeling prior to bleb creation has been recommended as a way to reduce the injection pressure required to create the bleb [28] and thus reduce reported RPE damage caused by higher flow injections; however, ILM peeling is not performed in clinical trials at this time as it may increase reflux from the subretinal space and be counterproductive.

Olufsen et al. also raised a question of interplay between the injection pressure and duration of holding the cannula in subretinal space, pointing to the surgeon’s tremor as one of the factors involved in postoperative retinal disruption [27]. Based on our institution’s experience with subretinal gene therapy delivery, other important factors to consider are assuring that the surgeon has excellent wrist support during the injection to minimize tremor and watching the depth of the cannula within the bleb to avoid mechanical RPE damage during the injection. We utilize a novel asymmetric wrist rest, which has been shown to reduce surgeons’ hand tremors. It ensures adequate support when operating on the patient’s same side as the dominant hand and on the contralateral side of the dominant hand instead of placing the hand on the patient’s forehead. This assures the highest level of surgeon hand support during subretinal injections [31].

If the goal is to include the fovea in the treatment, then the surgeon must be careful to observe the fovea with intraoperative OCT in order to avoid excessive foveal stretch and potential macular hole formation. Xue et al. showed that the foveal stretch depends on the injection volume and the distance of retinotomy from the fovea [12]. Based on their calculations, selecting retinotomy further away from the fovea allows us to reduce the foveal stretch, and ≥3 mm is ideal. This was corroborated by the findings of Sisk and colleagues, who showed that creating a retinotomy within 3.7 mm of the fovea, or approximately 2 disk diameters, and greater injection volumes were associated with a bullous foveal detachment, when the blebs extended posteriorly [32]. Since a macular hole is a highly undesirable complication, leading to reflux from the subretinal space and placing the patient at a risk of visual loss, the surgeons must be very careful to adjust their technique and utilize intraoperative OCT to monitor the fovea during the injection [20].

**Figure 5:**
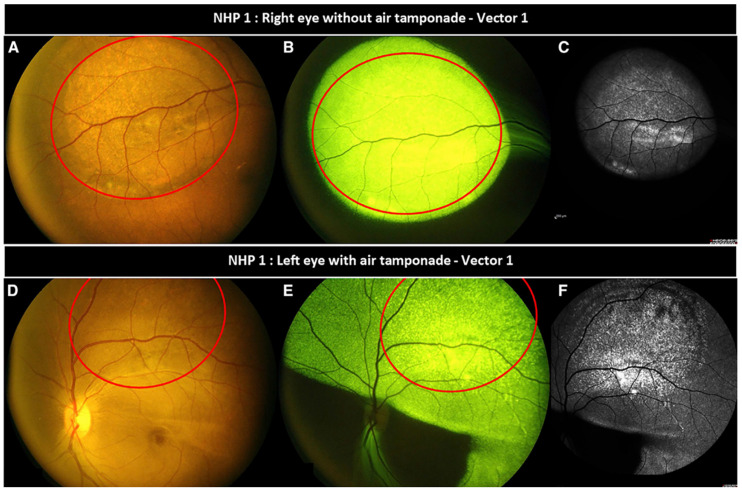
(**A**–**C**) Vector (unmodified AAV2) distribution is limited to the injection site area in the eye without air tamponade. (**D**–**F**) Vector distribution migrates beyond the initial bleb in eyes with post-injection air tamponade. The red circle represents the bleb distribution. Retrieved from Intravitreal air tamponade after AAV2 subretinal injection modifies retinal EGFP distribution [33].

After subretinal vector injection, some protocols include fluid-air exchange and air tamponade as is recommended in the surgical manual for the FDA-approved voretigene neparvovec-rzyl therapy [8]. Air–fluid exchange is thought to allow for a more complete removal of refluxed vector and as a way to stabilize the retina and help reoppose the retinal layers postoperatively. Ducloyer and colleagues recently explored the dynamics of intravitreal air tamponade in a non-human primate model [33]. The study found that intravitreal air strongly alters vector distribution in the subretinal space, driving vector expression well beyond the confines of the initial subretinal bleb [Figure 5]. In contrast, eyes without post-injection air tamponade saw target expression limited to the initial bleb [33]. Since predicting the exact migration of the bleb under air is difficult, caution is advised in employing this technique. On the other hand, this leads to the idea of using intravitreal air tamponade to gently push the bleb towards the fovea or to help it spread in order to treat a wider area of the retina, albeit at the cost of vector migration outside the intended target area. The latter effect may be particularly troublesome in patients where consistent post-surgical supine positioning is not feasible, leading to an even greater propensity for migration. Alternatively, Garg et al. describes a novel technique in a live porcine model which utilizes subretinal air to tamponade a bleb intraoperatively and post-surgically. In this case, the subretinal air creates a one-way mechanical support, allowing fluid to enter or be manipulated in the subretinal space while preventing unwanted reflux of vector or collapse of the bleb [34]. This has not been tried in non-human primates, and a potential effect of air causing RPE atrophy in this setting should be evaluated. A common strategy to prevent postoperative reflux is avoiding hypotony by suturing all sclerotomies and requiring the patient to recover for at least an hour in a flat supine position to avoid bleb migration.

### 3.4. Robot-Assisted Subretinal Delivery

One of the active areas of research in subretinal delivery is the development of robotic systems to assist or automate the injection. As mentioned above, manual subretinal injections are limited by physiologic human tremor, lack of tactile feedback, variability in applied forces, and difficulty maintaining slow, controlled movements over prolonged periods—all of which may contribute to inconsistent bleb formation or mechanical trauma to the retina. Robots can help minimize such shortcomings and enhance repeatability, especially as subretinal gene therapy becomes more widespread. A number of recent studies have evaluated this possibility [Table 2]. Maierhofer et al. compared subretinal injection efficacy using a custom-made robot versus manual surgery in 72 ex vivo porcine eyes [35]. Their custom robot was investigator-controlled, and intraoperative OCT was used to visualize the procedure, which employed a standard three-port PPV approach to inject perfluorocarbon subretinally. Higher incidence of successful bleb formation (73.7% vs. 61.8%) and lower incidence of intravitreal reflux (14.3% vs. 66.7%, *p* < 0.001) were observed in the robot-assisted group, with no significant difference in complication rates between groups [35]. Yang et al. also developed a custom subretinal surgery robot and evaluated its procedural efficacy compared to manual surgery [36]. Their custom robot was also fully investigator-controlled. The study used this platform to assist in delivering 1% sodium fluorescein to the subretinal space in porcine eyes. Conventional OCT, fundus photography, and video motion capture analysis were then employed to compare procedural efficacy of robotic versus manual delivery. The robot-assisted group saw decreased mean tremor amplitude (0.3681 vs. 18.8779 pixels, *p* < 0.0001) compared to the manual group, with no significant differences in subretinal fluid volume or subretinal cross-sectional area of the retinal bulge noted between groups, albeit the surgical duration was significantly increased in the robotic procedure [35,36].

Huang et al. utilized the intraOcular RoBotic Interventional System (iORBIS) robotic manipulator platform in combination with a novel predictive model for bleb formation in a gelatin retinal phantom model. Their method automated both cannula insertion and subretinal injection, achieving insertion angles unattainable by human surgeons and reflux-free bleb formation in all their twenty experiments [37]. Zhang et al. presented another autonomous method using the Steady Hand Eye Robot (SHER) with iOCT for automated high-precision needle navigation in subretinal injection. They validated the proposed method using a silicone eye phantom, as well as twenty ex vivo porcine eyes. The study reported a 100% success rate for subretinal injections in their ex vivo experiments, while maintaining scleral forces well under safety thresholds during navigation (<15 nM) [38]. Alternatively, Abid et al. introduced an integrated OCT probe within a 36G flexible subretinal injection cannula that enables a semi-automated guided approach to subretinal injection. Ex vivo validation of their novel instrument on porcine eyes revealed a 95% success rate in creating retinal blebs (95% CI: 83.1–99.4), with approximately 75% of injected volume transferring to the subretinal bleb (mean bleb volume 0.75 ± 0.23 µL) [39]. Dehghani et al. introduced “intelligent virtual B-scans” to rapidly process volumetric intraoperative OCT data to estimate instrument pose and automate injection trajectory in real-time based on a selected target point [40]. While the study does not demonstrate bleb formation, they achieve high navigation accuracy within ex vivo porcine eyes (32 ± 4 μm) [40]. Similarly, Arikan et al. present a novel “B5 scan” method which generates 3D reconstruction of surgical targets in real-time using iOCT. This allows for dynamic monitoring and adjustment of insertion position during injection, which they achieved with an error of 23 ± 13 µm during all 20 successfully created subretinal blebs [41]. Certainly, more development is needed to address shortcomings including slow latency times [42], limited translation to existing surgical tools, and injection backflow [40], especially before testing automation in humans. However, these advancements hold promise for enhancing surgical ease and precision.

**Table 2:** Studies evaluating robotic-assisted subretinal injection efficacy (2022–2025).

Study	Subject	Robot	Automated	Results	Limitations
Maierhofer et al. (2023) [35]	Ex vivo porcine eyes	Custom robot	No	- Higher incidence of successful bleb formation.	- Use of perfluorocarbon injection not fully representative of real gene therapy pharmaceuticals.
				- Lower incidence of intravitreal reflux.	
Yang et al. (2022) [36]	Ex vivo porcine eyes	Custom robot	No	- Significantly decreased mean tremor amplitude.	- Limited sample size per group (N = 5)
Huang et al. (2023) [37]	Retinal model	iORBIS robotic manipulator	Yes	- Reflux-free bleb formation in 100% of cases.	- Use of gelatin surrogate to model human retina. - Simplified regression formulae.
Arikan et al. (2025) [41]	Ex vivo porcine eyes	Steady Hand Eye Robot	Yes	- Real-time 3D reconstruction of tissue in relation to instrumentation. - 100% success rate of subretinal bleb creation.	- Focus on axial (Z) axis accuracy, potentially neglects horizontal motion error. - Use of open-sky approach in ex vivo porcine eyes.
Zhang et al. (2024) [38]	Silicone eye phantom; ex vivo porcine eyes	Steady Hand Eye Robot	Yes	- 100% success rate of subretinal injection in all ex vivo eyes. - All scleral forces significantly below reported safety threshold (<15 mN).	- Use of BIOM lens introduced image distortion and limits translation to human eyes.
Dehghani et al. (2023) [40]	Ex vivo porcine eyes	Steady Hand Eye Robot	Yes	- Rapid volumetric iOCT processing and automated instrument navigation.	- Limited/unclear sample size.
Abid et al. (2022) [39]	Ex vivo porcine eyes	Steady Hand Eye Robot	Yes	- 95% success rate of subretinal bleb formation.	- Large injection probe limits translation to clinical utility.

### 3.5. Novel Non-Vitrectomy Subretinal Approaches

**Figure 6:**
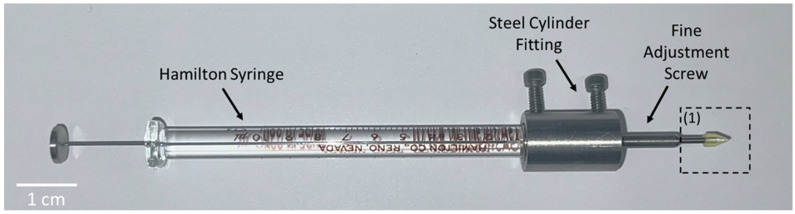
Novel transscleral microneedle injector. Retrieved from a non-surgical method for subretinal delivery by transscleral microneedle injection by Hejri et al. [43].

While the standard three-port PPV approach described above enables direct access to the SRS, its surgical complexity, complications, and cost have prompted recent investigation of alternative non-vitrectomy approaches to subretinal delivery. One such approach developed by Hejri et al. uses a transscleral microneedle to achieve minimally invasive delivery to the SRS [43] [Figure 6]. Their design features tailored microneedle length to match the thickness of the sclera and choroid and a vacuum probe to stabilize the eye. They demonstrated minimally invasive subretinal bleb formation in vivo in rat, mouse, and rabbit eyes, without retinal perforation, choroidal hemorrhage, or vitreous loss [43]. The injection procedure took around 1 min to perform per eye, compared to an approximate length of 60–90 min per eye in standard PPV surgeries [44]. Limitations of their study include lack of intraocular pressure measurement during or after injection, as well as a lack of anatomical confirmation during entry into the SRS itself. Further refinement of their design and eventual translation to human models offer greater delivery efficiency and the possibility of multiple injections as an in-office procedure [43]. Another study, by Xiangdong et al., used a two-port non-vitrectomized approach to deliver subretinal gene therapy in six patients with Bietti crystalline dystrophy (BCD) [44]. The operation times ranged from 9 to 14 min, bypassing core vitrectomy while directly advancing the injection cannula into the subretinal space under viscous fluid control (VFC) mode. Postoperative evaluation confirmed retinal reattachment and no subretinal fluid after 24 h in all six patients, with no adverse effects noted at the 9-month follow-up [45]. Although this report suggests safety of a non-vitrectomized surgical approach and faster operative times, there may be an increased risk of iatrogenic retinal breaks or detachment. In a separate study, Wood et al. reported a novel method to access the subretinal space without vitrectomy, using the nanovitreoretinal (NVR) subretinal gateway device (Vortex Surgical, Chesterfield, MO, USA). A 2-port pars plana approach is described, inserting a 25-gauge chandelier endoilluminator in one port and the NVR subretinal gateway device in another. The device needle is advanced into the mid-vitreous cavity, with subsequent very fine cannula extension to and through the retinal surface, creating a self-sealing retinotomy [46]. They report success using this method to clear and displace a subretinal hemorrhage in two patients undergoing subretinal injection of recombinant tissue plasminogen activator (tPA) [46]. Gyroscope Therapeutics utilized their Orbit Subretinal Delivery System (Orbit SDS) in conjunction with their GT005 gene therapy for treating dry age-related macular degeneration (AMD) with subretinal injection without a need for vitrectomy or retinotomy in phase 1/2 FOCUS trial (NCT03846193) [47]. This method offers another potential way to decrease the complications associated with vitrectomy and to minimize surgical times.

## 4. Suprachoroidal Delivery

### 4.1. Background

The suprachoroidal space (SCS) is the area located between the choroid and sclera, beginning at the scleral spur anteriorly with extension circumferentially and posteriorly. Emerging data has demonstrated the utility of this space for minimally invasive access for targeted delivery into the posterior segment of the eye. The rationale behind drug delivery to the SCS is selective, long-lasting administration to the retina, retinal pigment epithelium, and choroid with minimal drug exposure to the anterior segment of the eye. This strategic administration bypasses barrier layers such as the vitreous and ILM [48]. This results in greater bioavailability along the retina and choroid and mitigates risks associated with vitreous contact, such as retinal breaks, retinal detachments, and cataract formation [49,50]. Additionally, the SCS is readily expandable, with an ability to expand 7.5-fold without significant lasting effect on SCS anatomy [51]. One downside of this approach is that the SCS lies outside the blood–retinal barrier and thus is not immune-privileged; however, studies have demonstrated a lower humoral response after injection into SCS compared to the intravitreal space [48,49]. Importantly, the SCS can be readily accessed in an outpatient office without the need for a surgical procedure [50]. Together, the favorable pharmacokinetic features and outpatient accessibility may make SCS injections potentially advantageous for the clinician and patient alike.

**Figure 7:**
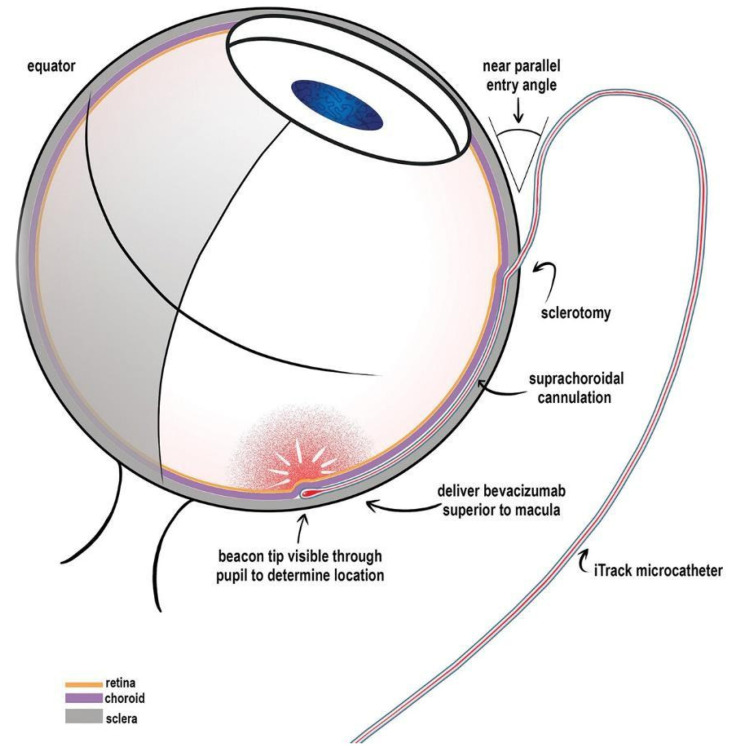
Schematic of iTrack microcatheter access to the suprachoroidal space. Retrieved from Alternative application of an iTrack microcatheter and canaloplasty: case report and literature review by Kicińska et al. [54].

**Figure 8:**
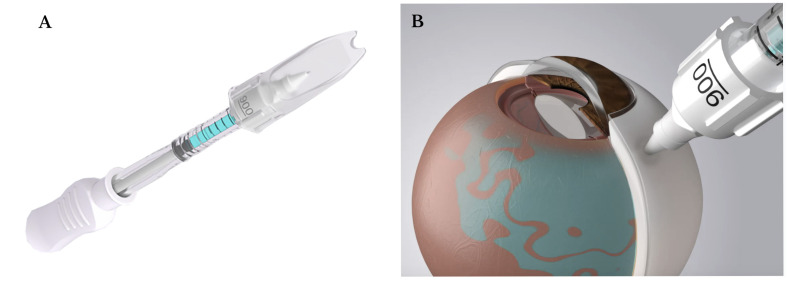
(**A**) Clearside suprachoroidal space (SCS) microinjector. (**B**) Illustration of suprachoroidal injection with the Clearside SCS microinjector. Retrieved from Clearside Biomedical, Inc. (Alpharetta, GA, USA). [57].

The SCS can be accessed via a microcatheter, a hypodermic needle, or a microneedle. Catheters, such as the 250 Angstrom iTrack microcatheter, can be inserted through a scleral incision and advanced posteriorly into the SCS, with confirmation of catheter placement using indirect ophthalmoscopy [52,53] [Figure 7]. This approach allows for direct visualization of delivery location (via illuminated catheter tip) and direct access to the posterior pole but requires an operating room environment [54]. The SCS can also be accessed via hypodermic needle, wherein the needle is inserted through the sclera behind the limbus with slow and controlled advance until a loss of resistance. While this approach utilizes readily available materials, it is heavily dependent on surgeon expertise with significant risk of injecting into unintended structures [55]. Microneedles, with needle lengths slightly longer than scleral and conjunctival thickness (900 µm or 1100 µm), are inserted perpendicular to the sclera until a loss of resistance is felt [56]. The injection will not proceed if the needle opening is still in the sclera, and the injectate is inserted over 5–10 s with compression over the conjunctiva to form a sealing gasket to minimize reflux. This enables an improved safety profile with ease of use in an outpatient clinical setting. A Clearside SCS microinjector is now FDA-approved for suprachoroidal injections and is utilized for triamcinolone acetonide (Xipere^®^, Alpharetta, GA, USA) injections for uveitis macular edema [57] [Figure 8].

### 4.2. Preclinical Developments

The investigation of suprachoroidal delivery of AAV vectors for gene therapy has garnered significant interest. Peter Campochiaro’s laboratory has been the leader in this field. The paper from his laboratory, by Ding et al., demonstrated that AAV8-mediated suprachoroidal gene transfer results in widespread ocular expression of green fluorescent protein in the eyes of rats, pigs, and nonhuman primates [21]. In rats, suprachoroidal injection of ABBV-RGX-314, an AAV8 vector expressing a ranibizumab-like anti-VEGF monoclonal antibody fragment (Fab), was widely distributed and resulted in widespread transgene expression throughout the RPE and photoreceptors cells with anti-VEGF Fab levels comparable to those achieved with subretinal injection, preventing VEGF-A-induced vascular leakage with similar efficacy [21]. The above study did not report adverse immunogenic effects in the animals tested; however, the inherent immunogenicity of viral vector delivery, particularly in non-immune-privileged spaces like the SRS, must be considered in future applications.

Non-viral vector-mediated gene therapy delivery into the suprachoroidal space is also being investigated, predominantly through lipid-based and polymer-based nanoparticles (LBNPs and PBNPs, respectively). Although LBNPs have not been used for retinal disease treatment suprachoroidally, the Poly(β-amino esters) (PBAEs) are a synthetic, biodegradable PBNP investigated for suprachoroidal injection. PBAEs facilitate efficient intracellular delivery of nucleic acids through endocytosis and endosomal escape. In rat eyes, suprachoroidal injection of PBAEs enabled sustained GFP expression in the retina for at least 6 months, with broader biodistribution than intravitreal or subretinal delivery, and, while achieving lower overall expression than AAV8, it could be enhanced with repeated injections [53]. More recently, studies in minipigs demonstrated that a single suprachoroidal injection of PBAEs enables broad and persistent retinal transfection in human-sized eyes without toxicity. Increasing dosages improved consistency and expression levels across multiple retinal locations, with sustained transgene expression over 12 weeks [58]. Importantly, as more evidence emerges on the efficacy of PBAEs, the choice of delivery route for gene therapy may be influenced. For example, PBAEs may be well suited to suprachoroidal administration, where their broad tissue distribution and repeat-dosing potential offer advantages over viral vectors. Conversely, the strong transduction efficiency and durability of AAVs make them more effective when localized expression is desired, such as with subretinal injections. Despite this success, however, much work needs to be done to optimize intracellular and intranuclear DNA delivery.

### 4.3. Clinical Trials

Drug delivery into the SCS has been leveraged in recent clinical studies for several retinal diseases, most notably with steroids and anti-VEGF agents. To demonstrate, PEACHTREE evaluated the efficacy and safety of suprachoroidally injected triamcinolone acetonide (CLS-TA) in 160 patients with macular edema secondary to noninfectious uveitis [59]. Treatment was well tolerated, and 47% of patients in the CLS-TA group achieved a gain of 15 or more letters in BCVA compared to 16% in the control group (*p* < 0.001). PEACHTREE and subsequent trials MAGNOLIA and AZALEA demonstrated low induced adverse effects, paving the way for FDA approval of Xipere in 2021 [60,61].

Ongoing clinical trials are investigating ABBV-RGX-314 in nAMD (AAVIATE trial) and diabetic macular edema (ALTITUDE trial). AAVIATE is an ongoing phase II, multi-center, open-label, randomized, active-controlled, dose-escalation trial at three dose levels (2.5 × 10^11^, 5 × 10^11^, and 1.0 × 10^12^ GC/eye) across six cohorts, including patients with and without AAV8 neutralizing antibodies. Interim data from 2024 shows mild to moderate intraocular inflammation and episcleritis occurred in some patients, particularly at higher doses, but resolved with topical steroids [62]. Patients who received prophylactic steroids had no cases of intraocular inflammation. At 6 months, changes in BCVA were minimal across doses (−2.8 to −2.2 ETDRS letters), while monthly ranibizumab improved BCVA by +4.0 letters. Retinal thickness changes varied, with minimal differences between groups. Fewer rescue anti-VEGF rescue injections were needed in ABBV-RGX-314-treated patients, with 50% of those receiving the highest dose requiring none. These patients maintained stable vision and excellent exudation control. Overall, ABBV-RGX-314 was well tolerated, with manageable inflammation and reduced need for anti-VEGF therapy. Interim data from the ALTITUDE trial evaluated ABBV-RGX-314 in DR patients with a diabetic retinopathy (DR) severity score (DRSS) of 47–65, without center-involving DME, and no recent anti-VEGF treatment [63]. Three dose levels (2.5 × 10^11^, 5.0 × 10^11^, and 1.0 × 10^12^ GC/eye) were tested and further sub-stratified by DRSS, along with 20 untreated controls. Inflammation was minimal, with mild episcleritis occurring more frequently at higher doses. At one year, among non-proliferative DR (NPDR) patients, a ≥2-step DRSS improvement was seen in 33% (dose 1), 20.8% (dose 2), and 12.5% (control). DR worsening was more common in controls (37.5%) than in treated groups. Notably, dose 2 arm patients experienced an 89% reduction in vision-threatening events compared to controls, highlighting ABBV-RGX-314’s potential for DR management.

## 5. Intravitreal Delivery

### 5.1. Background

Intravitreal injections have become a cornerstone of ocular therapeutics, especially for managing diseases of the posterior segment such as ARMD, DR, and IRDs. This technique involves the direct administration of pharmacologic or gene therapy agents into the vitreous cavity, enabling localized treatment with minimal systemic exposure. It is typically performed in an outpatient setting under sterile conditions using a 30- to 33-gauge needle inserted 3.5 to 4.0 mm posterior to the limbus, usually in the inferotemporal quadrant [64]. The procedure is rapid, generally well-tolerated, and highly amenable to repeated administration, making it ideal for chronic conditions requiring ongoing intervention.

The application of intravitreal injection for gene therapy emerged in the early 2000s as researchers sought alternatives to the technically demanding subretinal approach. In contrast to subretinal delivery, intravitreal injections offered a less invasive method, broader treatment area, and a well-established procedural safety profile. Initial studies demonstrated that adeno-associated virus (AAV) vectors delivered intravitreally could effectively transduce inner retinal layers, including ganglion and Müller glial cells, but showed limited efficacy in reaching the outer retina due to barriers such as the ILM [65]. Nevertheless, intravitreal delivery proved promising for diseases affecting inner retinal structures, such as Leber hereditary optic neuropathy (LHON), which became one of the main clinical applications of this method [66]. The success of early LHON trials validated the clinical feasibility of intravitreal gene therapy and spurred vector engineering efforts aimed at enhancing its retinal penetrance and specificity [66]. Multiple trials are not employing intravitreal viral vector delivery to treat IRDs.

### 5.2. Preclinical Developments

Building on this foundation, intravitreal therapy (IVT) has become a transformative strategy in ophthalmic gene therapy, particularly for inherited retinal degenerations (IRDs) [67]. While subretinal delivery remains standard for targeting photoreceptors and the RPE, its invasiveness and localized scope limit its utility [65]. Intravitreal injection provides broader retinal coverage, can be administered in an outpatient setting, and permits repeat dosing [68]. However, challenges persist. IVT-mediated gene delivery faces biological and immunological barriers, including poor vector diffusion across the ILM and increased host immune responses [68]. To address these issues, recent efforts have focused on developing engineered AAV capsids with enhanced tropism, non-viral delivery platforms, and refined injection protocol [69]. Although retinal transduction levels via IVT remain inferior to subretinal routes, ongoing technological innovations are narrowing this gap.

Preclinical studies are central to overcoming the anatomical and immunological challenges associated with intravitreal gene delivery, particularly the limited transduction of photoreceptors due to the ILM. Recent investigations have focused on capsid engineering and physical targeting. A peptide-modified AAV2 variant developed by Wubbins et al. demonstrated improved photoreceptor transduction and reduced humoral immune response following intravitreal delivery in murine models. This vector incorporated a cell-penetrating peptide to enhance ILM penetration and reduce immunogenicity [70]. Wang et al. recently introduced a magnetically guided AAV delivery platform using magnetic nanoparticles (MNPs) conjugated to AAV vectors in an ex vivo porcine retina model. With external magnetic field manipulation, spatially selective gene expression was achieved, offering a non-invasive method to bypass capsid tropism constraints [71]. Additionally, Zhou et al. reported the development of PT1, an AAV5-derived capsid variant that enhanced retinal transduction and exhibited resistance to preexisting neutralizing antibodies—an essential feature for repeat dosing in chronic conditions [72].

### 5.3. Clinical Trials

The translational progress of intravitreal gene therapy is evident in recent clinical trials targeting both inherited and acquired retinal diseases. A milestone was achieved in 2025 with the treatment of children with Leber congenital amaurosis (LCA) using intravitreal injection of an AAV vector carrying the AIPL1 gene. The intervention restored partial vision, allowing patients to recognize faces and navigate their environment [73]. AGTC-RS1-001 is a first-in-human, dose-escalation Phase I/II clinical trial evaluating the safety and efficacy of intravitreal delivery of rAAV2tYF-CB-hRS1, a recombinant AAV2 vector encoding the retinoschisin (RS1) gene, for the treatment of X-linked retinoschisis (XLRS) [74]. Conducted by Applied Genetic Technologies Corporation (AGTC), the study explores the feasibility of intravitreal administration to target retinal cells while avoiding the surgical risks of subretinal injection. The results demonstrated that the treatment was generally well tolerated but failed to show measurable efficacy, ultimately resulting in the halting of the study in 2018 [75], highlighting both the potential and limitations of intravitreal AAV delivery for inner retinal diseases. Intravitreal gene therapies are also under development for nAMD. Ixoberogene soroparvovec (Ixo-vec) delivers the aflibercept gene via an AAV vector, with Phase 1 trials demonstrating durable VEGF suppression and sustained visual acuity over 3 years, significantly reducing injection frequency [76]. Phase 3 is ongoing (NCT06856577). Similarly, 4D Molecular Therapeutics’ 4D-150 combines aflibercept expression with RNA interference against VEGF-C [77]. In Phase 2 studies, 77% of patients remained injection-free after 24 weeks, with an 89% reduction in annualized injection rates. Phase 2 for DME (NCT05930561) and phase 3 for AMD are ongoing (NCT07064759, NCT06864988).

## 6. Discussion

Emerging developments in the field of retinal gene therapy promise greater accuracy, flexibility, and the potential for reduced treatment burden. Despite this, limitations still exist, which may preclude widespread adoption of these developments. Regarding the subretinal approach, along with the inherent surgical risks and procedural cost of PPV, the treatment itself remains a significant barrier. The sole FDA-approved subretinal gene therapy voretigene neparvovec-rzyl (Luxturna, Spark Therapeutics, Inc., Philadelphia, PA, USA), for example, costs USD 425,000 per eye [78]. A cost-utility analysis for Luxterna reveals a large incremental cost [79]. Direct medical costs for Luxterna were about USD 1,039,000 versus USD 213,400 for standard care, producing an incremental cost-effectiveness ratio (ICER) of about USD 643,800/quality-adjusted life-years (QALY) [79]. Furthermore, if the therapeutic duration of these therapies is limited or unknown, then the financial assumption of a “one-time cure” is undermined. Many analyses assume durable effects but may need to model treatment degradation over time [80]. Integration of novel developments such as autonomous or robotic delivery to subretinal gene therapy, while promising increased accuracy and reproducibility, could add to this financial burden and reduce scalability. Conversely, office-based retinal gene therapy through suprachoroidal, intravitreal, and non-vitrectomizing subretinal delivery could alleviate such issues of cost, repeatability, and scalability. These approaches reduce the need for surgical infrastructure and staff, as well as operating room time, lowering both procedural and associated post-operative costs. Minimally invasive administration also facilitates repeat dosing, which may be necessary in treatments or vectors that degrade quickly. Furthermore, this simplified delivery could expand access to a broader patient population and increase the possibility of widespread clinical utility, particularly in settings with limited surgical resources. There are currently no FDA-approved suprachoroidal or intravitreal gene therapies for retinal disease; therefore, these potential advantages require ongoing investigation and may not be realized in the near future.

## 7. Conclusions

Significant efforts have been made to refine the delivery of retinal gene therapy. Subretinal delivery via PPV remains the gold standard for targeting photoreceptor and retinal pigment epithelium disease, including but not limited to Leber congenital amaurosis, retinitis pigmentosa, choroideremia, achromatopsia, X-linked retinoschisis, and Stargardt disease. Recent advancements in this technique aim to enhance safety and surgical reproducibility and improve precision. Autonomous and non-autonomous robot-assisted surgery is being explored to assist in critical steps of subretinal delivery, including bleb creation and vector injection. Non-vitrectomizing techniques for subretinal delivery are also being investigated, which could reduce treatment risks and decrease surgical time. These novel advancements are still in their infancy and have lacked robust integration into human eyes or significant in vivo applications. Furthermore, the major drawbacks of cost, surgical complexity, and limited duration of effect still preclude the widespread adoption of retinal gene therapy through a subretinal approach. These limitations underscore the need for continued development and refinement of delivery techniques. Intravitreal injections offer a less invasive approach by delivering viral vectors directly into the vitreous cavity, thereby facilitating outpatient administration, ability to treat larger retinal areas, and suitability for repeat dosing. Though physiological barriers like the ILM and immune responses against vectors limit transduction efficiency, advances in vector engineering and delivery strategies are aimed at addressing these challenges. Ongoing clinical trials and preclinical innovations are expanding their application across inherited and acquired retinal diseases, signaling a promising future for intravitreal gene therapy. Suprachoroidal delivery, a relatively novel route, involves injecting gene therapy vectors into the space between the sclera and choroid. It offers another promising route for targeted, minimally invasive product delivery into the posterior segment. Its advantageous pharmacokinetics, reduced immunogenicity compared to intravitreal route, and ease of outpatient access make it an attractive alternative to traditional approaches. Clinical trials have begun to demonstrate safety and efficacy, particularly for AAV-based therapies in retinal diseases. Work with nonviral vectors remains in infancy, but early studies show encouraging signs of broad retinal transfection. As optimization continues, the suprachoroidal space offers a scalable, low-immunogenic alternative for gene delivery. Collectively, these advancements highlight an overall shift toward safer, more precise, and less invasive delivery methods for retinal gene therapy. As procedural refinement and clinical validation progress, these surgical approaches are poised to expand treatment options for a broad range of inherited and acquired retinal diseases.

## Figures and Tables

**Table 1:** Overview of gene therapy vectors.

Vectors	Advantages	Disadvantages	References
Adenoviral (~1–3%)	- No integration into host genome	- More immunogenic	
	- High levels of expression within 24–28 h	- Rapid clearance	[3,5,6,7]
	- Large capacity (30 kB *)		
Adeno-associated (~80–90%)	- No integration into host genome - Low immunogenicity	- Limited capacity (4.7–5.0 kB)	[3,7]
	- Stable expression - Long-term expression - Broad tropism - High diffusion/penetration	- Slower transduction	
Lentiviral/Retroviral (~5–10%)	- Stable expression	- Integrate into host genomic DNA (possible off-target insertion, mutagenesis)	[3,7]
	- Long-term expression		
	- High transduction efficiency		
	- Large capacity		
Non-Viral (Liposomes, Polymers, Oligonucleotide, Nanoparticles) (1–2%)	- Large capacity	- Lower specificity	[5,7,8]
	- Less immunogenic	- Lower stability	

* kB = kilobase.

## Data Availability

The raw data supporting the conclusions of this article will be made available by the authors on request.
